# Examining structural brain changes in patients with PTSD following cognitive processing therapy

**DOI:** 10.3389/fpsyt.2026.1821652

**Published:** 2026-07-31

**Authors:** Annika Bernacki, Tiffany A. Kolesar, Ronak Patel, Sarah J. Chaulk, Iman Beheshti, Natalie Wright, Ji Hyun Ko

**Affiliations:** 1 Department of Human Anatomy and Cell Sciences, Rady Faculty of Health Sciences, University of Manitoba, Winnipeg, MB, Canada; 2 PrairieNeuro Research Centre, Kleysen Institute for Advanced Medicine, Health Science Centre, Winnipeg, MB, Canada; 3 Department of Clinical Health Psychology, Rady Faculty of Health Sciences, University of Manitoba, Winnipeg, MB, Canada; 4 Graduate Program in Biomedical Engineering, Price Faculty of Engineering, University of Manitoba, Winnipeg, MB, Canada

**Keywords:** cognitive processing therapy, diffusion tensor imaging, magnetic resonance imaging, posttraumatic stress disorder, voxel based morphometry

## Abstract

**Introduction:**

Posttraumatic stress disorder (PTSD) is a psychiatric condition occurring following exposure to psychological trauma. Neuroimaging studies have highlighted neuroanatomical differences in individuals with PTSD. Cognitive Processing Therapy (CPT) is an evidence-based frontline treatment for PTSD. Although CPT has shown success in decreasing clinical symptom severity, its underlying neural mechanisms remain unclear. We hypothesize that structural changes occur following CPT within key brain regions that have previously been linked to PTSD.

**Methods:**

Fifty-six participants, including patients with PTSD (*n* = 32) and healthy controls (*n* = 24), underwent T_1_-weighted structural magnetic resonance imaging (MRI) and diffusion tensor imaging (DTI) scans at baseline and follow-up. The PTSD group completed a twelve-week CPT treatment, while the controls did not undergo any treatment. To investigate the effects of successful CPT treatment, four participants who did not respond to treatment were excluded from analyses. T1-weighted images were analyzed using voxel-based morphometry and cortical thickness was estimated within pre-defined regions-of-interest. Fractional anisotropy, mean diffusivity, radial diffusivity, and axial diffusivity were estimated using DTI.

**Results:**

Significant interactions between group and time were observed in both grey matter (GM) and white matter (WM) volume analyses — decreased left thalamic GM volume and increased left cerebellar WM volume were observed in patients with PTSD, following CPT treatment. Furthermore, a significant interaction between group and time showed increased fractional anisotropy in the left cerebellum following treatment in the PTSD group. Cortical thickness and other DTI-related measures did not differ.

**Discussion:**

Our findings suggest that CPT leads to neuroanatomical changes in the thalamus and the cerebellum, and these may be associated with lasting effects of CPT-related benefits in PTSD.

## Introduction

Posttraumatic stress disorder (PTSD) is a psychiatric disorder which occurs after direct or indirect exposure to a psychologically traumatizing event (1). The stress response to trauma can have diverse impacts on the brain (2). Globally, 70.4% of individuals report having experienced a form of trauma in their lifetime (3). The lifetime prevalence of PTSD is 5.6% amongst trauma-exposed people (4). The first-line treatment for PTSD, mood, and anxiety disorders is cognitive behavioural therapy (CBT), which aims to establish links between thoughts, behaviours and emotions (5). Cognitive processing therapy (CPT) is a specialized form of CBT which focuses on reframing intrusive thoughts that have occurred following a traumatic experience (6). Specifically, this evidence-based, “gold standard” therapy (7) uses written exposures to reduce distress, which helps patients modify their maladaptive appraisals (8, 9). These maladaptive cognitions are often overaccommodated or assimilated within PTSD patients (10) and are typically referred to as “stuck points,” which are rigid, maladaptive beliefs about oneself, others or the environment that arise from trauma (11). CPT is typically delivered over a twelve-week period, once a week for 90 minutes, in either individual or group settings (12). Various meta-analytic reviews have shown that CPT is effective in reducing trauma-related distress (8, 10, 13). This reduction has been shown to persist at 5–10 year follow-ups (14), suggesting that neurobiological changes—potentially including structural plasticity—may contribute to sustained symptom improvement (15).

Historically, the prefrontal cortex, amygdala, and hippocampus are the brain regions most implicated in PTSD (16–18). Alterations in regional activity have been investigated using neuroimaging modalities such as MRI (19). A meta-analysis by Patel et al. reported consistent findings of reduced activation in medial prefrontal regions alongside heightened amygdala reactivity (19). Functional MRI (fMRI) data further revealed potential hyperactivation of the salience network, as well as altered patterns of engagement within the central executive and default mode networks (19). More recently, other regions of the brain such as the thalamus (20–22) and cerebellum (22–24) have been recognized for their functions related to PTSD. Alterations to these structures may alter the regulatory behaviours of an individual, including both cognitive function and emotional regulation (25). In the present study, structural MRI is used to further understand these changes. While functional alterations reflect dynamic neural responses, structural measures may index longer-term neuroplastic changes that could underlie sustained therapeutic effects (15).

Although CPT has been associated with clinically meaningful reduction in PTSD symptoms (26), the neurobiological mechanisms underlying these therapeutic effects remain incompletely understood. fMRI has been used previously to investigate CPT and revealed altered connectivity in PTSD patients (27); however, functional measures capture dynamic brain states and may not fully explain the durability of treatment response (15). To date, most neuroimaging studies evaluating structural brain changes in PTSD are basic science in nature (i.e., does brain structure in PTSD differ from HC); clinical trials (i.e., evaluating the effects of treatment on brain structure) are critically needed (28). In a study conducted by Yakemow et al. (2024) brain volume differences in PTSD (*n* = 40) vs. HC (*n* = 27) participants were assessed only prior to receiving CPT therapy. The authors found that there was reduced grey matter volume (GMV) in the mid-temporal lobe region of PTSD patients, a finding which parallels related research (29). Kroes et al. found a link between increased flashbacks and decreased volume in the medial temporal gyrus of PTSD patients (30). Alongside the hippocampus, the mid-temporal region of the cortex functions in memory, particularly within the re-experiencing of traumatic events (29). This suggests that alterations in temporal lobe structure may be involved in flashbacks that occur due to PTSD (30).

Structural neuroimaging may provide complementary insight into longer-term neuroplastic changes that could support sustained symptom improvement. Previous studies have identified gray and white matter abnormalities associated with PTSD symptom severity, yet the extent to which these structural features are modifiable following evidence-based psychotherapy remains unclear (31, 32). In the current study, T_1_-weighted MRI and diffusion tensor imaging (DTI) were used to examine gray and white matter structural changes, respectively, in PTSD patients following CPT, in comparison to healthy controls (HC). T_1_-weighted MRI was used for voxel-based morphometry (VBM) and cortical thickness (CT) analyses, which represent the level of atrophy and deformation. DTI was used to assess fractional anisotropy (FA), mean diffusivity (MD), axial diffusivity (AD) and radial diffusivity (RD), which represent the microstructural integrity of white matter (33). To our knowledge, this is the first study in adults to evaluate the neural anatomical changes associated with CPT in a community sample of individuals with PTSD.

We hypothesize that CPT will be associated with measurable structural changes in brain regions implicated in PTSD, reflecting neuroplastic processes related to improved cognitive and emotional regulation. Specifically, we expect patterns consistent with partial normalization or compensatory adaptation within corticolimbic and regulatory networks (e.g., prefrontal cortex, amygdala, hippocampus, thalamus, and cerebellum), when compared with HCs. By integrating multiple structural neuroimaging modalities, this study aims to advance understanding of the neural mechanisms that may support the therapeutic effectiveness and durability of CPT in PTSD.

## Materials and methods

### Participants

A total of 67 participants were recruited for our parent clinical trial investigating overall neural changes associated with a twelve-week CPT treatment intervention (Wright et al., under review). However, not all participants completed the study due to the onset of the COVID-19 pandemic, resulting in 56 participants with baseline and follow-up MRI data. Participants were sorted into PTSD (*n* = 32) and HC (*n* = 24) groups. The PTSD group consisted of treatment-seeking participants from the community and operational stress injury clinic (Winnipeg, MB, Canada) who had experienced a Criterion A traumatic event—trauma types were highly variable and were not amenable to grouping. Scores from the CAPS-5 (34) and PTSD Checklist-5 (PCL-5) (35) were used to characterize PTSD symptoms and severity, in accordance with the DSM-5. Exclusion criteria for PTSD participants included the presence of a neurological illness, as well as substance dependence and/or uncontrolled bipolar or psychotic disorder, as defined by the Mini International Neuropsychiatric Interview (MINI) (36). To investigate the effects of successful CPT treatment, participants from the PTSD group were excluded (*n* = 4) if they showed no change in CAPS-5 scores following treatment, or if symptoms worsened (i.e., CAPS-5 scores increased); these participants made up the non-responder (NR) group. The HC group consisted of trauma-exposed controls (TEC; *n* = 10) who had experienced a criterion A traumatic event but subthreshold symptoms (i.e., CAPS-5 total severity scores ≤ 5), along with trauma-naïve controls (TNC; *n* = 13). All control participants were pooled to make up the HC group due to low sample size, and none had any diagnosed psychiatric disorders. The HC group did not undergo any CPT intervention, while the PTSD group received twelve weeks of CPT (one 90-minute session/week), which was co-facilitated by a registered psychologist and psychology trainee/mental health clinician, who have been trained in the administration of CPT (37). Only PTSD participants who finished all CPT sessions were included in our study.

### Data acquisition

MRI data were obtained with a 3 Tesla Siemens MAGNETOM Verio IMRIS scanner (Erlangen, Germany) equipped with a 12-channel head coil. These images were collected at the Kleysen Institute for Advanced Medicine at the University of Manitoba, Bannatyne campus (Winnipeg, Manitoba). An MPRAGE sequence was used to acquire T_1_-weighted structural data with the following parameters: TR/TE/TI = 2300/3.02/900 ms; flip angle = 9°; field of view (FOV) = 256 mm x 256 mm with 1.00 mm x 1.00 mm x 1.00 mm resolution; 240 slices; interleaved acquisition. DTI data were acquired using a diffusion-weighted single-shot spin echo EPI sequence with the following parameters: TR/TE = 10,400/79 ms; flip angle = 90°; FOV = 240 mm x 240 mm with 2.5 mm x 2.5 mm x 2.5 mm resolution; 65 slices; slice thickness = 2.5 mm (no inter-slice gap); interleaved acquisition; 30 diffusion weighted images (b = 700 s/mm2); 5 reference images (b = 0 s/mm^2^). MRI data was obtained from two time points; baseline (2–4 weeks prior to treatment starting), and follow-up (within 2 weeks following the final CPT session; one participant was scanned 3 weeks after treatment ended).

### Voxel-based morphometry preprocessing and analysis

T_1_-weighted images were preprocessed in SPM12 (https://www.fil.ion.ucl.ac.uk/spm/), using the CAT12 toolbox (https://neuro-jena.github.io/cat/) in MATLAB (Version R2023a). Longitudinal segmentation (optimized for plasticity/learning effects) was performed through the pairing of baseline and follow up scans. The high-dimensional shooting template was used for registration; forward and inverse deformation maps were saved and modulation was applied. Images were then smoothed (FWHM = 4mm x 4mm x 4mm), estimated, and visualized using SPM12 and XJView (https://www.alivelearn.net/xjview) software. Factorial analyses were used to examine the effects of successful CPT treatment on GMV and WMV separately; interaction effects (group × time) were examined for both volume measures. Age, sex, years of education, and total intracranial volume (TIV) were used as covariates. A cluster forming threshold was defined at *p* < 0.001 (peak-level), with extent threshold corrected using a false discovery rate (FDR) at *q* < 0.05. GMV and WMV were extracted from any significant clusters for further *post-hoc* analyses (contrasting pre- vs. post- for each group) using the Statistical Package for the Social Sciences (SPSS, IBM Corp., version 27.0, Armonk, NY) software.

### Cortical thickness analysis

CT measurements were obtained through SPM12, using the CAT12 toolbox. The Desikan-Killiany-Tourville (DKT) atlas (38) was used and is made up of thirty-four pre-defined regions-of-interest (ROI) in each of the left and right cerebral hemispheres. These 68 ROIs were used to analyze CT changes across groups in pre- and post-CPT scans. Differences between groups were compared using general linear model (GLM) with age, sex, and years of education used as covariates.

### DTI analysis

In our subset of 52 HCs and PTSD responders, one was excluded from DTI analysis as no DTI data were available, resulting in 28 PTSD responders and 23 HCs. Probabilistic tractography was performed using SPM12 (https://www.fil.ion.ucl.ac.uk/spm/), ACID (http://www.diffusiontools.org/), and DTI&Fiber (https://www.uniklinik-freiburg.de/mren/research-groups/diffperf/fibertools.html) toolboxes in MATLAB (Version R2023a). Segmented structural T_1_-weighted images from the CAT12 analyses described above were used for spatially normalizing the DTI data. DTI data were converted from 4D images to 3D images and preprocessed using the ACID toolbox. Motion and eddy current correction were performed to ensure spatial alignment and reduce geometric distortions, followed by fitting of the diffusion tensor (robust tensor fitting algorithm; default values: normalization factor = 4, confidence interval = 0.3, residual smoothing = 16, regularization = 0.0001, smoothing kernel = 3 x 3 x 3). FA, MD, AD and RD were all examined—an average white matter mask created from all of the participants was applied during analysis. The same statistical approach was used in the VBM analysis.

### Statistical analysis

Statistical analyses were performed using SPSS software. Demographic data was statistically analyzed using independent and paired t-tests or chi-square tests in SPSS. Repeated-measures GLMs were used to examine the changes in CAPS-5 and PCL-5 scores from visit one to visit two, controlling for age, sex, and years of education.

## Results

### Demographics

Education significantly differed among HC and PTSD groups whereas sex and age did not (see **Table 1**). However, all three variables were used as covariates within our neuroimaging analyses. Details of clinical benefits will be published elsewhere (Patel et al., in preparation).

**Table 1:** Demographic data and mean (± SD) questionnaire scores for baseline (pre-treatment) and twelve-week follow-up (post-treatment, values in bold).

Questionnaire	PTSD	HC	*t* or *χ*^2^	df	*p*
*N*	32	24			
Age	40.2 (± 11.6)	33.7 (± 13.8)	1.884	54	0.065
Sex (M:F)	12:20	10:14	0.015	1	0.902
Education	13.9 (± 2.8)	16.4 (± 3.4)	3.027	54	0.003*
CAPS-5 pre-CPT treatment	32.2 (± 9.2)	0.8 (± 1.5)	16.475	54	<0.001*
**CAPS-5 post-CPT treatment**	**18.0 (**± **11.4) ^a^**	**0.3 (**± **0.6)**	**7.570**	**54**	<0.001*
PCL-5 pre-CPT treatment	43.7 (± 13.3)	2.3 (± 2.9)	14.893	54	<0.001*
**PCL-5 post-CPT treatment** **Medication (N)** ** Anxiolytics** ** Antidepressants** ** Antipsychotics** ** Mood stabilizers** ** Stimulants** ** Cannabis**	**27.3 (**± **17.3) ^a^** **23** **8** **14** **3** **3** **0** **5**	**2.0 (**± **2.8)** **0** **0** **0** **0** **0** **0** **0**	**7.075**	**54**	<0.001*

Data are presented for chi-squared analysis (i.e., sex) or independent t-tests contrasting PTSD vs. HC.

^*^ Significant after Bonferroni correction for multiple comparisons. CAPS-5, Clinician-Administered PTSD Scale for DSM-5; PCL-5, PTSD Checklist. ^a^paired t-test from baseline, p < 0.001.

### CAPS-5 and PCL-5 scores

The CAPS-5 and PCL-5 scores were significant for the main effect of time (CAPS-5: *F*(1,52) = 8.996, *p* = 0.004; PCL-5: *F*(1,52) = 9.613, *p* = 0.003), group (CAPS-5: *F*(2,52) = 95.036, *p* < 0.001; PCL-5: *F*(2,52) = 8.950, *p* < 0.001), and group × time interaction effects (CAPS-5: *F*(2,52) = 33.456, *p* < 0.001; PCL-5: *F*(2,52) = 82.085, *p* < 0.001). *Post hoc* analyses indicate that the CAPS-5 and PCL-5 scores were significantly different pre-and-post intervention in the PTSD group (**Table 2**). These were corrected for multiple comparisons using a Bonferroni *p* < 0.05 threshold.

**Table 2:** *Post-hoc* analysis evaluating differences in CAPS-5 and PCL-5 score from each group at visit 1 and visit 2.

Questionnaire	Mean difference	*p*
CAPS-5 score		
HC group PTSD group NR group	0.522 (1.621) 16.857 (1.469) -4.000 (3.887)	0.749 <0.001* 0.308
PCL-5 score		
HC group PTSD group NR group	0.304 (3.041) 17.643 (2.757) 8.000 (7.293)	0.921 <0.001* 0.278

The mean difference (standard error) was calculated using visit 1 minus visit 2.

*Significant at the *p* < 0.001 level.

CAPS-5, clinician-administered posttraumatic stress disorder scale for DSM-5.

PCL-5, posttraumatic stress disorder checklist-5.

NR, Non-responder.

### VBM analysis—grey matter

**Figure 1:**
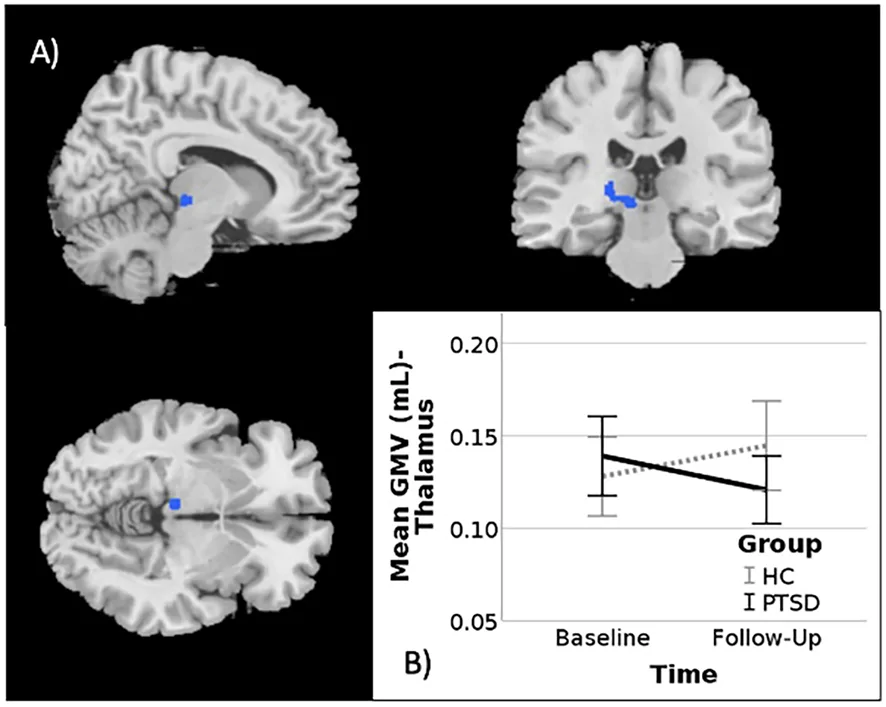
**(A)** Grey matter volume significantly differed in the thalamus (blue). **(B)** The interaction of group × time was significant (cluster-defining threshold *p* < 0.001, corrected for multiple comparisons at FDR *q* < 0.05, with *post hoc* analyses revealing grey matter volume decreased over time in the PTSD group.

Using VBM, a significant group × time interaction was observed for GMV using the HC and PTSD responder groups. Age, sex, years of education and TIV were all controlled for as covariates. A significant cluster (*F*(1,96) = 21.81; *p* = 0.012, FDR corrected; 176 voxels; x = -9, y = -26, z = -4) was identified in the thalamus (see **Figure 1**); *post hoc* analyses revealed that GMV decreased over time within the thalamus cluster in PTSD patients (mean difference = -0.028, CI = 0.010–0.046, *p* = 0.003) while it was increased in HC (mean difference = 0.025, CI = 0.006–0.045, *p* = 0.013).

### VBM analysis—white matter

**Figure 2:**
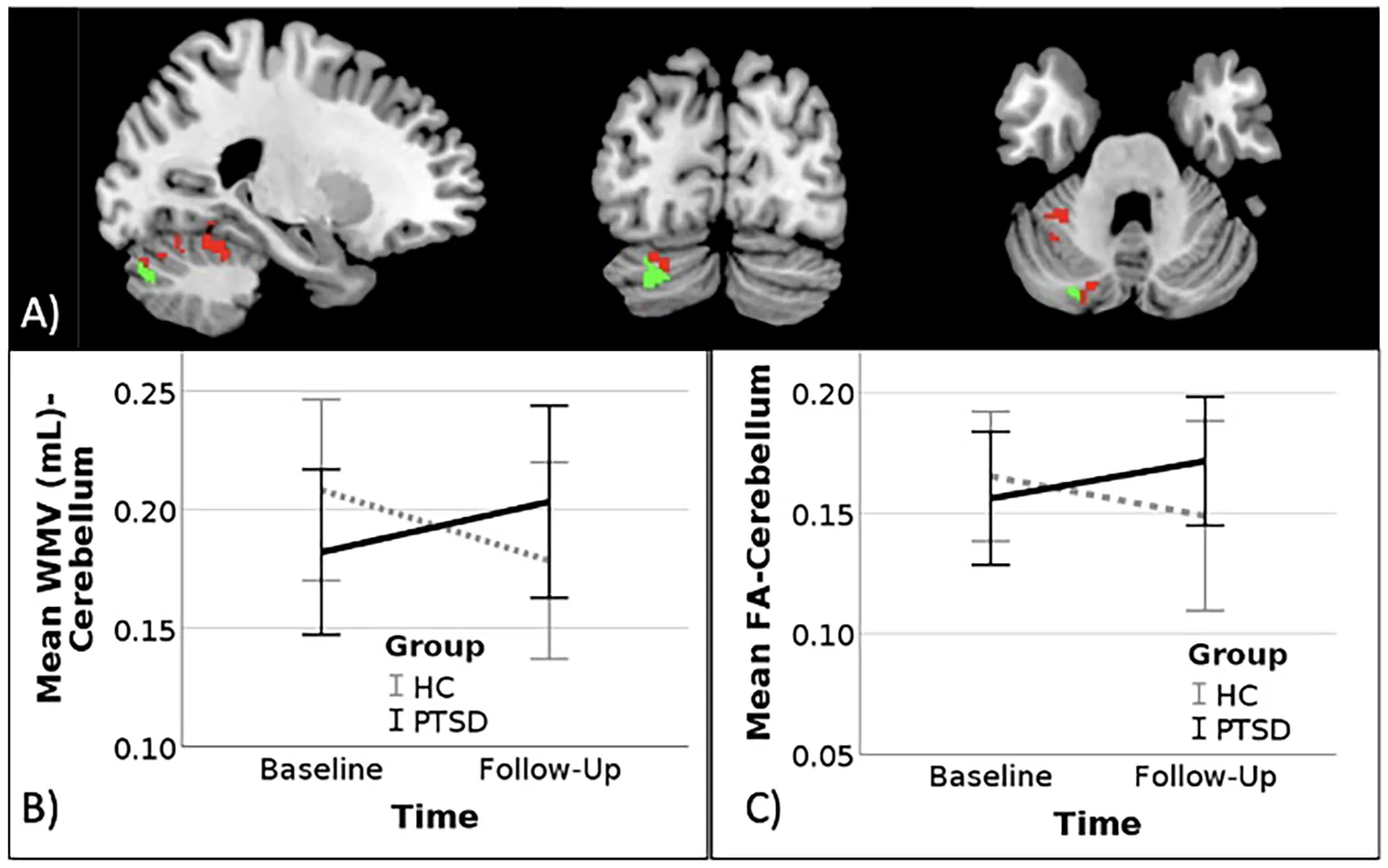
**(A)** Significant white matter changes in the cerebellum (white matter volume shown in green [crus I/II]; fractional anisotropy shown in red). **(B)** White matter volume significantly differed in the cerebellum for the interaction of group × time (cluster-defining threshold [CDT] *p* < 0.001, corrected for multiple comparisons at FDR *q* < 0.05). *Post hoc* analyses revealed white matter volume increased over time in the PTSD group. **(C)** Fractional anisotropy significantly differed in the cerebellum for the interaction of group × time (CDT *p* < 0.05, corrected for multiple comparisons at FDR *q* < 0.05). *Post hoc* analyses revealed fractional anisotropy increased over time in the PTSD group.

Using VBM, a significant group × time interaction was also observed for WMV using the HC and PTSD responder groups. Age, sex, years of education and TIV were all controlled for as covariates. A significant cluster (*F*(1,96) = 21.81; *p* = 0.040, FDR corrected; 253 voxels; x = -27, y = -78, z = -34) was identified in the posterior lobe of the left cerebellum (crus I/II; see green cluster in **Figure 2**); *post hoc* analyses revealed that WMV increased over time within the cerebellum cluster in PTSD patients (mean difference = 0.022, CI = 0.000–0.045, *p* = 0.048) while it was decreased in HC (mean difference = -0.031, CI = 0.007–0.055, *p* = 0.011).

### DTI analysis

DTI data were used to examine FA, MD, AD and RD. FA results showed a significant group (PTSD responder vs. HC) × time (pre vs. post) interaction (cluster defining threshold *p* < 0.05), while controlling for age, sex and years of education in the cerebellum (*F*(1,95) = 10.88; *p* = 0.013, FDR corrected; 920 voxels; x = -18, y = -46, z = -16; see red cluster in **Figure 2**). *Post hoc* analyses revealed that FA increased over time within the identified cluster in PTSD patients (mean difference = 0.015, CI = 0.007–0.023, *p <* 0.001) while it was decreased in HC (mean difference = -0.016, CI = 0.007–0.025, *p* = 0.001).

### Cortical thickness

No significant CT differences were observed between the HC and PTSD groups, following CPT treatment.

## Discussion

This novel study provides a deeper understanding of the neuroanatomical changes which occur in PTSD patients following successful CPT treatment: we observed reduced thalamic GMV and increased left cerebellar FA and WMV (crus II) following successful treatment (**Figures 1**, **2**). Basic science research has shown decreased cerebellar WMV in patients with PTSD compared to HC, with significant effects in the left crus II and left lobule VIIB (24, 39, 40). Furthermore, in a recent large scale (*N* = 4,215) VBM study, researchers identified reduced GMV and WMV in the cerebellum, including in crus II for patients with PTSD, compared to HC (40). These cerebellar GMV and WMV findings were also found in a large-scale meta-analysis, with emphasis in the left cerebellar hemisphere for GMV (24). Interestingly, these results were still observed when PTSD was compared to TEC, suggesting the differences were due to PTSD itself, rather than trauma-exposure. Coupled with the current clinical trial, these data suggest that successful twelve-week CPT intervention may work to reverse WM atrophy within these regions and begin to normalize brain structure. Importantly, this further suggests that white matter within this region can undergo neuroplastic adaptations following relatively brief behavioral interventions (39). Indeed, structural changes in GMV and WMV following short interventions have previously been documented (41, 42). For example, Taubert et al. (2012) reviewed multiple studies showing both GMV and WMV changes in response to skill acquisition, and that these structural adaptations correlate with behavioral improvements (42). The present study highlights the potential for CPT to induce meaningful neuroplasticity and contribute to long-term improvements in mental health.

The role of the cerebellum in mental health research has been significantly underappreciated in the past, primarily because it has historically been regarded as the hub for neuromotor regulation (43, 44). Cerebellar contributions were believed to be limited to coordinating and fine-tuning movements, overshadowing its potential involvement in broader aspects of mental health and cognitive functions (43). This narrow focus has resulted in a lack of comprehensive exploration into the influence of the cerebellum on emotional regulation, cognitive processes, and overall mental well-being (43). To understand the full extent of the role of the cerebellum, it is crucial to recognize that the cerebellum is extensively interconnected with other regions throughout the brain including stress-related regions including the hippocampus and amygdala (40). These connections may influence PTSD symptoms by interfering with normal brain-mediated stress responses through cerebro-cerebellar circuits (40). CPT allows individuals to alter fear conditioning and processing, potentially through the increase of WMV in the cerebellum (6). This finding was reinforced through our DTI analysis, as FA values increased following successful treatment in the neighboring regions of the left cerebellum (see **Figure 2**). This may indicate increasing white mattery integrity within the left cerebellum and strengthened structural connections with the cortex (45).

Cortical projection from the cerebellum to the cortex is mediated through the thalamus, another structure implicated in the present study. The thalamus has been recognized for its functions beyond acting as a sensory hub, including the regulation of awareness and cognition (20). Due to reciprocal pathways between the thalamus and amygdala, visual and auditory stimuli are relayed from the thalamus to the amygdala, allowing for the initiation of a fear response (46, 47). PTSD can cause increases in thalamic activation, including connectivity with the amygdala, which may be responsible for flashbacks and triggers (20). Although the thalamic changes induced by PTSD are not uniformly agreed on, emotional dysregulation within PTSD patients may be caused by hyperactivation of the thalamus, causing the person to relive traumatic experiences (48). In our study, we observed a decrease in GMV in the left thalamus following successful treatment in PTSD. Indeed, a prior study examining eye movement desensitization and reprocessing (EMDR) therapy also found decreased GMV in the left thalamus of patients with PTSD after treatment (48). These patients also showed decreased re-experiencing symptoms after EMDR therapy. Taken together with our findings, alterations in left thalamic gray matter volume may act as a critical neural substrate or marker for symptom improvement or normalization following psychological interventions in PTSD. One hypothesis is that the observed decrease in GMV reflects pruning of maladaptive neuroplasticity, which may have developed in response to chronic trauma (49). This structural refinement could reduce unnecessary or hyperactive synaptic connections, potentially lowering metabolic demand within the thalamus. In other words, the thalamus after therapy may become more functionally efficient, with decreased overactivation, which could contribute to improved regulation of sensory processing and fear responses (50). However, it should be noted that investigations of whole-thalamus volume in PTSD have been inconsistent—meta-analysis tends to show smaller thalamic volume in PTSD, with the exception that high comorbidity with major depressive disorder is correlated with higher thalamic volume (51). Additionally, severity of symptom clusters (e.g., re-experiencing vs. hyperarousal) may be more relevant to thalamus size than overall symptom severity (51)—the present parent clinical trial observed improvements in both re-experiencing and hyperarousal [Wright et al., under review]. Further investigations should be conducted to clarify the causal mechanisms underlying these structural changes and their ability to predict treatment outcomes. This may be possible through the identification of biomarkers that allow clinicians to stratify patients based on their likelihood of benefiting from CPT and support the development of more targeted interventions. CPT may potentially help to normalize thalamic responses, thereby contributing to improved emotional regulation and overall mental health (8).

These neuroanatomical findings in the cerebellum and thalamus can be understood within the broader clinical framework of trauma-related psychology. PTSD is characterized by disruptions in emotion regulation, increased threat sensitivity, maladaptive cognitive processing, cognitive distortions, and affective symptoms such as sensory hyperactivity, even in the absence of threat (52, 53). CPT directly targets these dysfunctional beliefs and responses, reducing the psychological stress response to them. Some evidence shows that by altering maladaptive thoughts and beliefs through CPT, activation of the hypothalamic-pituitary-adrenal axis (the body’s primary driver of stress) reduces, as evidenced by improvements in heart rate variability (54). This modification is rooted in the cognitive processing theory which suggests that when new, inconsistent information is processed, either assimilation or accommodation occurs (55). The central aim of CPT is to help patients replace inappropriately assimilated or overaccommodated beliefs (55). The observed structural changes in the cerebellum may reflect improvements to a bottom-up control over an overactive thalamus, caused by the cognitive restructuring learned via CPT. These cerebellar WM changes may result in better control over the thalamus, reducing the sensory flooding and hyperarousal often experienced in PTSD.

Extending this further, acute psychiatric responses following traumatic exposure highlight the extensive interaction between psychological trauma and neurobiological stress systems (56). These responses often involve heightened stress reactivity, which may contribute to long term changes in structure and function of the brain (57). High adversity populations such as veterans and war-exposed individuals/refugees typically have higher rates of PTSD than other traumas (58), and are often less amenable to treatment (59). Prompt intervention is necessary—perhaps successful early treatment in this group prior to the development of PTSD could help mitigate the neurological changes and symptomology associated with PTSD. Analyzing PTSD within the context of a broader trauma spectrum provides context for the interpretation of trauma-focused interventions in facilitating adaptation.

Interestingly, in our interaction analysis between time (pre- vs. post-) and group (PTSD vs. HC), we did not observe altered volume in the medial temporal region, which has often been observed to be relevant to PTSD symptoms (29, 30). Indeed, we have previously reported brain volume differences in the temporal regions in this sample of PTSD participants (*n* = 40) vs. HC (*n* = 27) at baseline (prior to intervention) (18). This discrepancy may be because while this PTSD cohort does have reduced volume in this region for the PTSD group (18), successful treatment was not dependent on regaining volume in this region, which was in line with a previous study that found a lack of change within this region following CBT (60). Instead, it is likely the case that improvement in PTSD symptom severity resulted from an alternate, compensatory pathway in more flexible brain regions that were able to structurally re-organize, via psychosocial intervention.

Our CT analysis did not reveal any significant differences when examining the group x time interaction. These outcomes may suggest there is a parallel trend in cortical thinning across both groups, or may simply be explained by the fact that because CT investigates changes in cerebral cortex, it is unlikely to see any change when structural differences are within the thalamus or cerebellar volume (61). It is equally important to consider that the variability in CT might be influenced by the choice of atlas utilized. Specifically, the DKT atlas employed in our study has been fine-tuned for the cerebral cortex but lacks rigorous validation and uses large parcellations, which may have been unable to detect small changes in thickness.

A puzzling observation was that all changes in PTSD were accompanied with changes in the opposite direction in HC. These participants were recruited from the community at large, and no intervention was provided. The scan interval was twelve weeks, and thus the observed structural changes in HC are likely be within the unspecific fluctuation of measurement error, rather than to other factors such as age. A recent computer simulation study suggested a nuanced approach is necessary when interpreting *post hoc* results in control groups following a significant voxel-wise interaction effect due to Berkson’s paradox (62). Berkson’s paradox occurs when independent data appear to be negatively correlated, due to the way data is selected. In low-powered neuroimaging studies, “selection” occurs when a significant cluster (observed during a voxel-wise interaction) is chosen for *post hoc* testing, which can result in spurious, opposite findings in the control group, compared to the treatment group (62). The interaction effect identifies regions of maximal difference, thus, only voxels with highly different values will be identified in the interaction. When treatment effects are weak, random noise within the significant region in the HC group have already been “selected” based on their maximally different signals, introducing a spurious negative correlation. In fact, in the simulation study (62), and the present data, a voxel-wise paired t-test within the HC group examining pre and post data did not reveal any significant changes (*p* < 0.05, FDR-corrected), indicating the opposite findings to the PTSD group are likely due to noise. This simulation study highlights that this paradox should not hinder the overall validity of the findings, rather, significant effects in the control condition may suggest that the overall statistical analysis was underpowered, i.e., there may be more false negatives while positive intervention effects are still valid (62).

Limitations of the present study include the small sample size which may have increased the likelihood of detecting false positive changes in HC, as well as limited the generalizability of these results. However, this “likely” false positivity in HC should not deter the interpretation of significant changes occurring in PTSD patients. Additionally, the HC group included individuals both with and without Criterion A trauma exposure. This introduces heterogeneity within the control group that may contribute to attenuated differences between the PTSD and HC groups. However, a recent large-scale VBM meta-analysis (*N* = 3507) observed that differences in GMV and WMV were highly similar whether the PTSD group was compared to trauma-exposed or trauma-naïve control groups (24). In any case, future research should be done with large sample sizes and multi-site datasets in order to improve statistical power and allow for stratification of variables (i.e., trauma-exposure in controls, trauma-type in PTSD groups, responders vs. non-responders, etc.). Second, although longitudinal changes were interpreted as reflecting therapy-related neuroplasticity, structural MRI measures are subject to short-term neurobiological fluctuations which are possible even in the absence of intervention (63). While the inclusion of a control group helps to mitigate this concern, it is complicated by the fact that opposing directions of change are observed in HCs, although as discussed, this may be due to Berkson’s Paradox. It is worthwhile to reiterate that the increase of cerebellar WMV in the PTSD group is in line with the literature—reduced cerebellar volume is often observed in PTSD while our results show that CPT shifts volume towards normalization. Our results are further strengthened by the use of a more conservative ‘whole-white matter’ analysis, rather than a region-of-interest analysis targeting the cerebellum. Another limitation is that the FA results are exploratory in nature (i.e., cluster-defining threshold *p* < 0.05); however, they align well with the more robust WMV changes observed in the cerebellum (see **Figure 2**), lending credibility to their likelihood of being true effects. Finally, the exclusion of treatment non-responders may limit the generalizability of these findings to all individuals with PTSD; future work should investigate neural differences between these groups to identify differences that may underlie the success of CPT treatment.

Overall, through VBM analysis we observed a reduction in GMV in the left thalamus in our PTSD group following treatment, possibly reflecting changes in thalamic function associated with trauma processing and recovery. Additionally, our results indicated an increase in WMV and FA in the cerebellum of PTSD patients following successful treatment, suggesting alterations in white matter structure and integrity are associated with improving resilience and reducing PTSD symptom severity. These results highlight the potential for CPT to effect rapid and significant improvements in symptom severity, but also pave the way for further research into the neuroanatomical mechanisms underlying these changes and their implications for long-term recovery in PTSD patients.

## Data Availability

The data that support the findings of this study are not publicly available due to ethical and privacy restrictions related to participant confidentiality. De-identified data may be made available from the corresponding author upon reasonable written request and subject to approval by the principal investigator, institutional ethics requirements, and applicable data-sharing agreements.
