# Correction: Examining structural brain changes in patients with PTSD following cognitive processing therapy

**DOI:** 10.3389/fpsyt.2026.1980013

**Published:** 2026-09-22

**Authors:** Annika Bernacki, Tiffany A. Kolesar, Ronak Patel, Sarah J. Chaulk, Iman Beheshti, Natalie Wright, Ji Hyun Ko

**Affiliations:** 1 Department of Human Anatomy and Cell Sciences, Rady Faculty of Health Sciences, University of Manitoba, Winnipeg, MB, Canada; 2 PrairieNeuro Research Centre, Kleysen Institute for Advanced Medicine, Health Science Centre, Winnipeg, MB, Canada; 3 Department of Clinical Health Psychology, Rady Faculty of Health Sciences, University of Manitoba, Winnipeg, MB, Canada; 4 Graduate Program in Biomedical Engineering, Price Faculty of Engineering, University of Manitoba, Winnipeg, MB, Canada

**Keywords:** cognitive processing therapy, diffusion tensor imaging, magnetic resonance imaging, posttraumatic stress disorder, voxel based morphometry

Author Sarah J. Chaulk was omitted as an author in the published article. The correct author list reads:

“Annika Bernacki, Tiffany A. Kolesar, Ronak Patel, Sarah J. Chaulk, Iman Beheshti, Natalie Wright and Ji Hyun Ko”

The **Author Contributions Statement** has been corrected to read:

“AB: Writing – original draft, Writing – review & editing. JK: Investigation, Project administration, Software, Supervision, Writing – review & editing. TK: Writing – review & editing. RP: Writing – review & editing. SJC: Investigation, Writing – review & editing. IB: Writing – review & editing. NW: Data curation, Writing – review & editing”.

The original version of this article has been updated.

